# Response of Source-Sink Characteristics and Rice Quality to High Natural Field Temperature During Reproductive Stage in Irrigated Rice System

**DOI:** 10.3389/fpls.2022.911181

**Published:** 2022-07-05

**Authors:** Debao Tu, Yang Jiang, Akram Salah, Mingli Cai, Wei Peng, Lijuan Zhang, Chengfang Li, Cougui Cao

**Affiliations:** ^1^National Key Laboratory of Crop Genetic Improvement, MOA Key Laboratory of Crop Physiology, Ecology and Cultivation (The Middle Reaches of Yangtze River), Huazhong Agricultural University, Wuhan, China; ^2^Rice Research Institute, Anhui Academy of Agricultural Sciences, Hefei, China

**Keywords:** temperature, reproductive stage, grain filling dynamics, source-sink characteristics, rice quality

## Abstract

Global warming greatly affects the development of rice at different growth stages, thereby deteriorating rice quality. However, the effect of high natural field temperature during reproductive stages on rice quality is unclear. Thus, grain filling dynamics, source-sink characteristics and quality-related traits were compared between two contrasting natural field temperature conditions namely normal (low temperature) (LRT) and hot (high temperature) growth season (HRT) during reproductive stage. Compared with LRT, HRT significantly increased chalky grain rate (about 1.6–3.1%), chalkiness level (about 4.7–22.4%), protein content (about 0.93–1.07%), pasting temperature, setback, and consistence, and decreased total starch content (about 4.6–6.2%). Moreover, HRT significantly reduced the leaf area index (LAI, about 0.54–1.11 m^2^ m^–2^), specific leaf weight (SLW, about 1.27–1.44 mg cm^–2^) and source-sink ratio (leaf-sink ratio and/or stem-sink ratio), shortened the active grain filling period by 3.1–3.2 days, and reduced the rations of dry matter translocation to grain (RDMs). In conclusion, we suggested that significant reduction in assimilate translocation after flowering, resulting in the reduced active grain-filling duration and the poor rice quality (high chalkiness and the poor eating and cooking quality), modulated by source-sink characteristics in response to high natural field temperature during reproductive stage. These results enriched the study of high temperature-stressed rice and served as an important reference for selecting high-quality, heat-tolerant varieties and protecting rice quality under high-temperature conditions.

## Introduction

Rice (*Oryza sativa L*.) is a staple food crop for more than half of the world’s population, but rice yield and quality are often reduced by high-temperature (Yang et al., 2020, 2021). Such drastic elevation in temperature is occurring more frequently in China (Deng et al., 2015). Large reductions in rice quality caused by high temperature stress were documented, resulting in serious economic losses (Liang et al., 2011; Shi et al., 2016a). Moreover, it is predicted that the global average surface temperature will rise by 1.0–3.7°C by the end of 2100 (IPCC, 2013). Thus, it’s necessary to comprehensively study the effect of high temperature on rice quality to ensure food security.

Rice quality is a complex agronomic trait, including the milling quality, appearance quality, eating and cooking quality and nutritional quality (Zhang et al., 2016; Zhen et al., 2019; Lal et al., 2022). Recently, the Rapid Visco-Analyzer (RVA) method is widely used to evaluate the eating and cooking quality (Jing et al., 2016; Kato et al., 2019; Yao et al., 2019). Numerous studies stated that high temperature during grain filling stage decreased milled rice rate, increased chalkiness, reduced total starch and amylose content, elevated protein content, and altered the RVA profiles of rice flour (Zhang et al., 2016; Yao et al., 2019). However, most of these studies focused on the effect of high temperature on rice quality during grain filling stage.

Reproductive stage is the period of flag-leaf emergence and rapid panicle growth (Wu et al., 2016). Previous studies stated that the high temperature during reproductive stage adversely reduced dry matter production, non-structural carbohydrates (NSC) remobilization, seed-setting rate, grain number and grain weight (Jagadish et al., 2014; Wu et al., 2016; Ali et al., 2019; Zhen et al., 2020). These findings suggested that the high temperature during reproductive play a fundamental role in rice growth and formation of rice quality. In spite of several controlled-temperature studies showed that the high temperature during the reproductive stage increased protein content, inhibited starch deposition and altered physicochemical characteristics of starch, most of these studies were conducted under controlled temperature chambers (Zhen et al., 2019; Lin et al., 2020). This approach limited in that it does not necessarily reproduce field conditions (Huang et al., 2013). Thus, it is necessary to find out the effect of high natural field temperature during reproductive stage on rice quality.

Moreover, the mechanism of the effect of high temperature during reproductive stage on rice quality is unclear. Grain filling involves the transport and synthesis of carbohydrates, proteins, and lipids in seeds. It has been proved that the mechanism of the effect of high temperature during grain filling stage on rice quality was contributed by reduced maximum and mean grain filling rate, which induced by reduction in assimilates translocation after flowering and modulated by source-sink characteristics (Shi et al., 2013, 2016b). In fact, assimilates generated either during grain filling (post-flowering) or redistributed from the reserve pool of the vegetative tissues (pre-flowering) determine successful grain filling in rice (Yang and Zhang, 2006). Moreover, previous studies indicated that heat stress is detrimental to plant development affecting photosynthesis, carbohydrate accumulation, starch synthesis, sucrose synthesis and transport, and photoassimilate accumulation in sink tissues, which ultimately affect the source-sink relationship (Shi et al., 2016b; Lal et al., 2022). According to these studies, we hypothesized that the effect of high temperature during reproductive stage on rice quality is also associated with grain filling dynamics, modulated by source-sink characteristics. Hence, in the present study, grain filling dynamics, source-sink characteristics and rice quality-related traits were compared between two contrasting natural field temperature conditions during reproductive stage, to reveal the differences of these and their association in response to high natural field temperature during reproductive stage.

## Materials and Methods

### Plant Materials, Site Description, and Growth Conditions

The same field experiments were conducted in 2017 and 2018 on Xiaowan Village (N32.0°, E112.8°) in Hubei province. The Hubei province located at the center of the Yangtze River Valley, has a humid subtropical climate, one of the most representative climates of the Yangtze River Valley. Temperature in this region have a similar change tendency in rice growth season, namely, it increases from April to June, reaches a peak in July and August, and declines thereafter from September to November. Three regional commercial japonica rice cultivars with wide planting area in central China namely Nanjing-9108 (NJ9108), Jing-565 (J565), and Jing Liangyou-4466 (J4466) were used in this study. They have excellent rice quality, but they were susceptible to high temperature. The cultivars were arranged in a randomized complete block design with three replications. The properties of soil were alluvial sandy loam with containing: pH = 6.66, organic matter = 34.3 g kg^–1^, total N = 1.58 g kg^–1^, available P = 8.75 mg kg^–1^and available K = 56.6 mg kg^–1^.

The rice growth temperature conditions can be divided into two scenarios (scenario A and B) in this study. In scenario A, there were two contrasting natural field temperature conditions during reproductive stage (jointing-heading), namely hot (high temperature) growth season (HRT) and normal (low temperature) growth season (LRT) during reproductive stage. The daily temperature during reproductive stage of HRT was continuous 0.5–5.6°C higher than that of LRT (Supplementary Figure 1). The mean daily temperature during reproductive stage of HRT was 29.7 (J565) and 29.9°C (NJ9108), which was 0.6 and 0.7°C higher than that of LRT, while the temperature during grain filling was similar in scenario A (Supplementary Table 1). In scenario B, there were two contrasting natural field temperature conditions during grain filling stage (heading-physiological maturity) i.e., hot (high temperature) growth season (HGT) and normal (low temperature) growth season (LGT) during grain filling stage. The daily temperature during grain filling stage of HGT was continuous 0.7–9.8°C higher than that of LGT (Supplementary Figure 1). The mean daily temperature during grain filling stage of HGT was 25.8 (J565) and 26.6°C (J4466), which was 1.6 and 3.1°C greater than that of LGT (Supplementary Table 1). In addition, the mean daily temperature during the first 20 days of grain filling stage of HGT was 29.1 (J565) and 28.3°C (J4466), which was 1.3 and 2.0 °C higher than that of LGT (Supplementary Table 1). The data of the experiments could be divided into two scenarios including eight samples J565-HRT, J565-LRT, NJ9108-HRT, NJ9108-LRT, J565-HGT, J565-LGT, J4466-HGT, and J4466-LGT.

In addition, field management was carried out according to local practices and was the same between the two years. The normal fertilization level (270 kg hm^–2^ total N, 135 kg hm^–2^ P_2_O_5_, 270 kg hm^–2^ K_2_O) was chosen in this study (Tu et al., 2020). Fertilizer-N was applied in four splits: 40% as basal, 20% at tillering, 20% at panicle initiation, and 20% at two weeks after panicle initiation. Fertilizer-P was fully applied as basal, and fertilizer-K was applied in two splits of 50% as basal and 50% at panicle initiation. The fertilizers were used in the forms of compound fertilizer, urea, calcium superphosphate, and potassium chloride. The field was flooded after transplanting, and a floodwater depth of 3–5 cm was maintained until a week before maturity except that the water was drained at maximum tillering stage to reduce unproductive tillers.

### Determination of Dry Matter and Leaf Area at Heading and Maturity Stage

Twelve plant samples from each plot were collected randomly (with the three outermost rows removed to minimize the border effect) at the heading and maturity stage. Plants were separated into leaves, stem-sheaths and panicles, and rice plant height, tiller number, leaf area, leaf, panicle and stem-sheath dry weight were recorded.

### Determination of Grain Filling Dynamics in Rice

The determination method of grain filling dynamics referred to the method of Zhu et al. (1988). When the rice was heading, the panicles which were heading at the same time were marked. Samples were taken every four days from heading to maturity, and 3 panicles were taken as a sample and repeated three times. Count the number of panicles, then thresh them, put them in an oven at 80°C and dry them to a constant weight. The average rice grain weight (mg grain^–1^) minus the hull weight was calculated, and the grain filling dynamics were fitted according to the Richards equation.

### Measurement of the Appearance Quality, Total starch Content, Amylose Content and Pasting Properties of Rice

Mature rice was threshed after harvest, air-dried, and stored at room temperature for 3 months until testing (Zhang et al., 2016). The appearance quality of rice was measured and analyzed with a scanner (Epson Expression 1680 Professional, Epson, America) and image analysis software (Chen et al., 2013).

The total starch was determined by the optical rotation method, and the amylose content was determined by the iodine blue colorimetric method (Sandhu and Singh, 2007). The protein content was determined by Kjeldahl method. RVA rapid viscosity analyzer (produced by Newport Scientific Instruments Co., Ltd., Warriewood, NSW Australia) was used to determine the viscosity characteristics of the starch, and the matching software TWC was used for analysis (Zhang et al., 2016). For the RVA procedure, 3.0 g samples of milled rice flour were mixed with 25 mL of deionized H_2_O, and the pasting programed cycle was set at 12.5 min. Starch samples were first heated at 50°C for 1 min and then heated from 50 to 95°C at a heating rate of 11.84°C per min. The temperature was held at 95°C for 2.5 min, and the starch was then cooled down to 50°C at a cooling rate of 11.84°C per min, and held at 50°C for 1.4 min. RVA characteristic values include peak viscosity, trough viscosity, final viscosity, breakdown (peak viscosity- trough viscosity), setback (final viscosity-peak viscosity), consistence (final viscosity- trough viscosity), and pasting temperature (Chen et al., 2021).

### Statistical Analysis

Analysis of variance (ANOVA) was used to analyze the differences in rice quality, source-sink characteristics and dry matter translocation. Data analysis was performed by using SPSS 21 and Microsoft Excel 2010. The graphs were prepared by using SigmaPlot 14.0.

## Results

### Differences of the Appearance Quality, Total Starch Content, Amylose Content in Response to High Natural Field Temperature

In scenario A, compared with J565-LRT, the protein content, chalky grain rate, chalkiness level and pasting temperature of J565-HRT separately increased by 0.93%, 1.6%, 4.7%, and 5.3°C, and the total starch content decreased by 4.6% (Table 1). Similarly, the protein, chalky grain rate, chalkiness level and pasting temperature of NJ9108-HRT separately increased by 1.07%, 3.1%, 22.4% and 6.5°C, and the total starch content decreased by 6.2%. These results indicated that high natural field temperature during reproductive stage significantly affected the rice quality. In scenario B, results showed that the chalky grain rate and chalkiness level of J565-HGT were 39.9% and 25.8% higher than that of J565-LRT, respectively. While the amylose content of J565-HGT was 1.8% lower than that of J565-LRT. For J4466-HGT, results also showed that the chalky grain rate and chalkiness level separately increased by 8.0%, 1.2%, and amylose decreased by 1.3%. The protein content of the two rice varieties showed slight differences between HGT and LGT. In addition, the trends of total starch content and pasting temperature of the two varieties were the same. Both high natural field temperatures during reproductive stage and during grain filling stage significantly damaged rice quality, but there were some differences in the changes of rice quality induced by them. Hence, it is necessary to understand the effect of high natural field temperature during reproductive stage on rice quality clearly.

**TABLE 1:** Difference of rice quality between two contrasting natural field temperatures conditions.

Scenario	Treatment	Protein content (%, w/w)	Total starch content (%, w/w)	Amylose content (%, w/w)	Chalky grain ratio (%)	Chalkiness level (%)	Pasting temperature (°C)
A	J565-HRT	7.59 ± 0.30a	71.5 ± 0.6c	15.9 ± 0.1a	81.5 ± 2.8b	41.3 ± 2.1b	81.2 ± 0.5a
	J565-LRT	6.66 ± 0.13b	76.1 ± 0.2b	16.0 ± 0.2a	79.9 ± 4.6b	36.6 ± 1.9c	75.9 ± 1.4b
	NJ9108-HRT	7.18 ± 0.30a	71.4 ± 1.2c	10.4 ± 0.2b	92.9 ± 2.9a	63.1 ± 2.4a	77.8 ± 0.9b
	NJ9108-LRT	6.11 ± 0.37c	77.6 ± 0.8a	10.4 ± 0.2b	89.8 ± 1.3a	40.7 ± 2.1b	71.3 ± 0.9c
b	J565-HGT	6.66 ± 0.13a	76.1 ± 0.2a	16.0 ± 0.2b	79.9 ± 4.6a	36.6 ± 1.9a	75.9 ± 1.4a
	J565-LGT	6.80 ± 0.09a	76.8 ± 0.4a	17.8 ± 0.4a	40.0 ± 4.3b	10.8 ± 1.7b	73.1 ± 0.5b
	J4466-HGT	6.07 ± 0.05b	74.5 ± 0.5b	15.2 ± 0.2c	12.6 ± 0.8c	2.3 ± 0.3c	71.2 ± 0.8*c*
	J4466-LGT	5.90 ± 0.33b	76.2 ± 0.4a	16.5 ± 0.2b	4.6 ± 0.3d	1.1 ± 0.2d	69.9 ± 0.5c

*HRT and LRT indicate hot (high temperature) and normal (low temperature) growth season during reproductive stage, respectively. HGT and LGT mean hot (high temperature) and normal (low temperature) growth season during grain filling stage, respectively. Different letters indicate that significant difference within the same column at P < 0.05. J565, NJ9108 and J4466 mean Jing 565, Nanjing 9108, and Jing liangyou 4466, respectively.*

### Variations of Pasting Properties of Rice Flour Under High Natural Field Temperature

This study also determined the RVA characteristics of rice (Table 2). There were significant differences in the RVA characteristic values of scenario A. The value of breakdown was high in all temperature conditions, and it showed some different changes of two varieties in response to HRT. However, the setback and consistence which were negatively associated with eating and cooking quality, demonstrated a significant increasing in response to HRT, and the changes of the two varieties were consistent. These results suggested that high natural field temperature during reproductive stage markedly deteriorated eating and cooking quality. However, there were slight changes of them in response to HGT, except the breakdown of J 4466 and the consistence of J565. In addition, the RVA characteristic values of scenario B performed slighter changes, compared with scenario A.

**TABLE 2:** Difference of RVA profile of rice starch between two contrasting natural field temperatures conditions.

Scenario	Treatment	Breakdown (cP)	Setback (cP)	Consistence (cP)
A	J565-HRT	2116.0 ± 35.2a	−388.0 ± 88.6a	1728.0 ± 53.5a
	J565-LRT	1759.0 ± 125.5b	−615.5 ± 53.0b	1025.7 ± 37.6b
	NJ9108-HRT	2168.3 ± 153.5a	−1142.0 ± 98.7c	1026.3 ± 45.8b
	NJ9108-LRT	2231.3 ± 107.2a	−1570.0 ± 122.5*d*	661.3 ± 26.3c
B	J565-HGT	1759.0 ± 125.5c	−615.5 ± 53.0a	1025.7 ± 37.6b
	J565-LGT	1748.5 ± 12.0c	−627.7 ± 7.5a	1125.0 ± 7.0a
	J4466-HGT	2225.7 ± 79.1a	−1177.7 ± 121.7b	1048.0 ± 46.4b
	J4466-LGT	2071.0 ± 30.0b	−1032.5 ± 27.5b	1032.7 ± 10.5b

*HRT and LRT indicate hot (high temperature) and normal (low temperature) growth season during reproductive stage, respectively. HGT and LGT mean hot (high temperature) and normal (low temperature) growth season during grain filling stage, respectively. Different letters present significant difference within the same column at P < 0.05. J565, NJ9108 and J4466 mean Jing 565, Nanjing 9108, and Jing liangyou 4466, respectively.*

### Effects of High Natural Field Temperature on Source-Sink Characteristics and Dry Matter Transport

**FIGURE 1:**
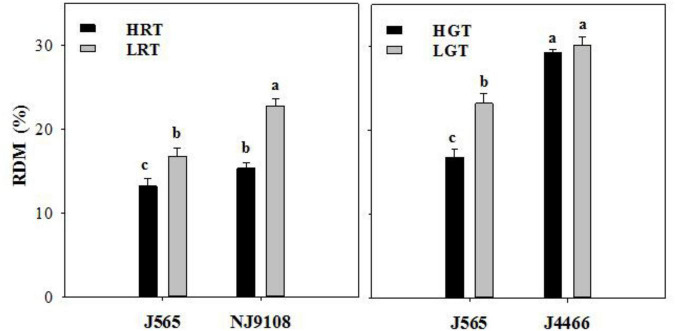
Difference in the ration of dry matter translocation to grain between two contrasting natural field temperature conditions in two scenarios. RDM: Ration of dry matter translocation to grain. Different letters present significant difference according to the LSD (0.05). HRT and LRT indicate hot (high temperature) and normal (low temperature) growth season during reproductive stage, respectively. HGT and LGT mean hot (high temperature) and normal (low temperature) growth season during grain filling stage, respectively. J565, NJ9108 and J4466 present Jing 565, Nanjing 9108, and Jing liangyou 4466, respectively. Different letters indicate significant difference at *P* < 0.05.

The source-sink characteristics demonstrated significant differences among different temperature conditions (Table 3). In scenario A, the leaf area indexs (LAIs) and specific leaf weights (SLWs) of LRT at heading stage were significantly higher than those of HRT. The LAIs of J565-LRT and NJ9108-LRT were 0.54 m^2^m^–2^and 1.11 m^2^m^–2^ higher than that of J565-HRT and NJ9108-HRT, respectively. In addition, the SLWs of J565-LRT and NJ9108-LRT were 1.44 mg cm^–2^ and 1.27 mg cm^–2^higher than that of J565-HRT and NJ9108-HRT, respectively. The source-sink ratio (leaf/sink ratio and stem-sheath/sink ratio) also displayed some differences. Obviously, the high natural field temperature during reproductive stage significantly affected source-sink characteristics. In scenario B, the LAIs and SLWs of J565-HGT at heading stage were higher than those of J565-LGT. Likewise, the LAI and SLW of J4466-HGT at heading stage were also higher than those of J4466-LGT. In addition, the stem-sheath/sink ratios of J565-HGT and J4466-HGT were significantly higher than that of J565-LGT and J4466-LGT, respectively. The leaf/sink ratio of J4466-HGT was also significantly higher than that of J4466-LGT. Moreover, the transfer efficiency of dry matter from the stem-sheath to grain also donated some differences among different temperature conditions (Figure 1). In scenario A, the rations of dry matter translocation to grain (RDMs) of J565-HRT and NJ9108-HRT were significantly lower than that of J565-LRT and NJ9108-LRT. These results suggested that the changes in source-sink characteristics sharply hindered the movement of assimilate to the panicle in response to high natural field temperature during reproductive stage. In addition, the RDMs of J565 and J4466 also decreased under HGT.

**TABLE 3:** Difference of characteristic of rice source and sink between two contrasting natural field temperatures conditions.

Scenario	Treatment	LAI (m^2^m^–2^)	SLW(mg cm^–2^)	Leaf/sink ratio (cm^2^ g^–1^)	Steam-sheath/sink ratio (mg g^–1^)
A	J565-HRT	6.23 ± 0.05b	5.03 ± 0.09b	49.8 ± 1.5a	530.3 ± 19.1a
	J565-LRT	6.77 ± 0.25a	6.47 ± 0.15a	42.2 ± 2.7b	511.1 ± 13.8*ab*
	NJ9108-HRT	6.32 ± 0.05b	5.03 ± 0.19b	42.4 ± 0.2b	473.9 ± 33.9b
	NJ9108-LRT	7.43 ± 0.81a	6.30 ± 0.09a	44.5 ± 2.4b	549.1 ± 17.2a
B	J565-HGT	6.77 ± 0.25c	6.47 ± 0.15a	42.2 ± 2.7b	511.1 ± 13.8b
	J565-LGT	6.30 ± 0.25c	5.43 ± 0.52b	43.6 ± 3.9b	373.7 ± 12.0c
	J4466-HGT	8.16 ± 0.01a	5.52 ± 0.07b	61.9 ± 4.3a	615.6 ± 24.8a
	J4466-LGT	7.40 ± 0.11b	5.37 ± 0.05b	36.4 ± 0.9c	334.2 ± 5.1*d*

*LAI, leaf area index; SLW, specific leaf weight. Different letters denote significant difference within the same column according to the LSD (0.05). J565, NJ9108 and J4466 mean Jing 565, Nanjing 9108, and Jing liangyou 4466, respectively. HRT and LRT indicate hot (high temperature) and normal (low temperature) growth season during reproductive stage, respectively. HGT and LGT mean hot (high temperature) and normal (low temperature) growth season during grain filling stage, respectively.*

### Dynamic Changes in Rice Grain Filling Under High Natural Field Temperature

**FIGURE 2:**
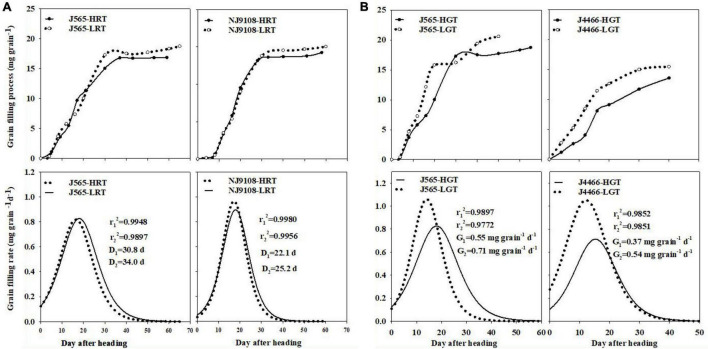
Grain filling process and grain filling rate of rice under different natural field temperature conditions in two scenarios. **(A)** is for scenario **(A)**; **(B)** is for scenario **(B)**. HRT and LRT indicate hot (high temperature) and normal (low temperature) growth season during reproductive stage, respectively. HGT and LGT mean hot (high temperature) and normal (low temperature) growth season during grain filling stage, respectively. J565, NJ9108 and J4466 present Jing 565, Nanjing 9108, and Jing liangyou 4466, respectively. Different letters denote significant difference at *P* < 0.05.

There were some differences of the grain filling dynamics among different temperature conditions (Figure 2). In scenario A, the durations of active grain filling of J565-LRT and NJ9108-LRT were 3.2 and 3.1 days longer than that of J565-HRT and NJ9108-HRT, respectively. In scenario B, the maximum grain filling rate of LGT was significantly higher than that of HGT. The mean grain filling rates of J565-LGT and J4466-LGT were 0.16 and 0.17 mg gain^–1^higher than that of J565-HGT and J4466-HGT, respectively. These changes were closely associated with the variations of rice quality in response to high natural field temperature. However, there were some differences in the changes of grain filling induced by HRT and HGT. This result indicated that there were certain differences in the mechanism of rice quality deterioration caused by them. Therefore, it’s important to find out the mechanism of rice quality deterioration caused by high natural field temperature during reproductive stage.

## Discussion

### Differences of Rice Quality in Response to High Natural Field Temperature During Reproductive Stage

Although the rice quality is mainly determined by genetic factors, external environmental factors also have an important influence on rice quality, especially temperature (Zhang et al., 2016; Lin et al., 2020). As the results of this study, there were significant differences of rice quality under different temperature conditions. In scenario A, compared with LRT, HRT significantly increased chalkiness and protein content, and decreased total starch content. These results were consistent with the findings that high temperature during reproductive stage increased chalkiness and protein content and inhibited starch deposition (Sulaiman et al., 2018; Zhen et al., 2019; Lin et al., 2020). Furthermore, HRT significantly increased pasting temperature, setback and consistence which have highly negative correlation with eating and cooking quality (Chen et al., 2021). This result may be attributed to the increased protein content and the decreased total starch content. As previous reported suggested that in whole grain structure starch granules interact with protein and other polysaccharides (Jing et al., 2021), which represented that the endogenous factors other than starch, such as starch granule-associated proteins may also significantly affect pasting properties (Zhang et al., 2016). Similarly, it proved that the elevated protein content and the decreased total starch content significantly declined the eating and cooking quality through the N fertilizer experiment (Huang et al., 2020). Thus, the accumulation balance of storage proteins and starches may play an important role in determining eating and cooking quality. In addition, the accumulation balance of storage proteins and starches coordinately regulated the formation of grain chalkiness (Tang et al., 2018). Therefore, we suggested that high natural field temperature during reproductive stage damaged the accumulation balance of storage proteins and starches, and ultimately induced the poor eating and cooking quality and the high chalkiness.

Numerous studies reported that high temperature during grain filling stage significantly increased the chalkiness and reduced the eating and cooking quality (Jing et al., 2016; Shi et al., 2016a; Zhang et al., 2016). In this study, results also demonstrated that chalky grain rate and chalkiness level significantly increased under HGT. The reason for these results was that HGT significantly decreased amylose content. It was consistent with the finding that heat and drought stress significantly induced high chalkiness because of the declined amylose content (Gao et al., 2012). However, there were slight variations of protein content, total starch content and the RVA characteristic values under HGT. These went against the previous studies that high temperature during the grain filling stage significantly increased the rice protein content and reduced the total starch content and the eating and cooking quality (Liang et al., 2011; Lin et al., 2016; Yao et al., 2019). There results mainly attributed to the fact that LGT accompanied by high natural field temperature during reproductive stage, and HGT accompanied by low natural field temperature during reproductive stage in the present study (Supplementary Table 1), while there were the same temperature conditions during reproductive stage in previous studies. This also indicated that high natural field temperature during reproductive stage strongly affected the eating and cooking quality.

### Source-Sink Characteristics in Response to High Natural Field Temperature During Reproductive Stage

Source-sink characteristics play an import role in determining rice quality (Shi et al., 2013; Chen et al., 2019). In the present study, results suggested that HRT damaged the source-sink characteristics, including the reduced LAI, the decreased SLW and/or the reduced source-sink ratio (leaf/sink ratio and stem-sheath/sink ratio). This might be induced by the significantly early heading under high temperature during reproductive stage (Ishii et al., 2011; Krishnan et al., 2011; Ali et al., 2019), as same as result donated in the present study (Supplementary Table 1). The short time for tissues and organs to acquire photoassimilates under high temperatures resulted in fewer and/or smaller organs and less biomass accumulation (Krishnan et al., 2011). As a result, these affected the leaf photosynthetic and the source-sink relationship (Lin et al., 2005). Likewise, there were also significant differences of source-sink characteristics in scenario B (LGT was lower than HGT). This might be attributed to the fact that LGT accompanied by high natural field temperature during reproductive stage, and HGT accompanied by low natural field temperature during reproductive stage in the present study (Supplementary Table 1). Furthermore, previous studies proved that the damaged source-sink characteristics significantly induced poor rice quality (Shi et al., 2013; Chen et al., 2019). Thus, we suggest that high natural field temperature during reproductive stage damaged the source-sink characteristics including the reduced LAI, the decreased SLW and the reduced source-sink ratio (leaf/sink ratio and stem-sheath/sink ratio), resulting in the poor rice quality.

### Grain Filling Dynamic in Response to High Natural Field Temperature During Reproductive Stage

It has been acknowledged that the carbohydrate allocation plays an important role in determining rice yield and quality, which is affected by heat stress (Shi et al., 2013; Zhang et al., 2018; Zhen et al., 2020). This study found that HGT significantly decreased the RDMs of J565 and J4466. This is mainly due to the inhibition the activity of key enzymes of starch synthesis in grains (Fu et al., 2016; Smith et al., 2018). Moreover, HRT also significantly declined the RDMs of J565 and NJ9108. This might be attributed to the damage of source-sink characteristics (Chen et al., 2019). Previous studies have stated that the RDM played an important role in determining rice grain filling (Yang and Zhang, 2006; Shi et al., 2017). It was no doubt that the maximum grain filling rate of LGT was significantly higher than that of HGT in scenario B. Interestingly, there was no the obvious difference of the maximum grain filling rate in scenario A, but the duration of active grain filling was quite different. Results showed that HRT significantly shortened the active grain filling duration. Numerous studies suggested that these were closely associated with the formation of rice quality (Jin et al., 2007; Huang et al., 2013; Shi et al., 2017; Zhao et al., 2019). Therefore, we suggested that high natural field temperature during reproductive stage significantly damaged source-sink characteristics, which hindered the movement of assimilate from stem-sheath to the panicle and shortened the active grain filling duration, and eventually induced poor rice quality (including the high chalkiness and the poor eating and cooking quality).

It is well known that temperature is related to other meteorological factors such as solar radiation and rainfall (Huang et al., 2013). Numerous evidences demonstrated that radiation had a positive impact on rice growth and development (Peng et al., 2004; Deng et al., 2015). However, this study showed that the weak rice growth and the poor rice quality were existed under high solar radiation. This result may be attributed to the fact that there were confounding effects of temperature and radiation (Deng et al., 2015; Tu et al., 2020). High temperature always accompanied with high solar radiation (Supplementary Table 2), and the former have significantly negative effects on rice growth (Krishnan et al., 2011; Ali et al., 2019; Lin et al., 2020). Furthermore, it is considered that temperature is the most important climatic factor governing the rice growth and the formation of rice quality (Chen et al., 2013; Huang et al., 2013; Zhang et al., 2016). Therefore, in the present study, the differences in rice growth and rice quality were mainly attributed to the temperature changes.

## Conclusion

In practice, each stage of the whole rice growth season may suffer from high temperature, especially reproductive stage and grain filling stage. This study showed that both HGT and HRT significantly affected rice quality, but there were some differences in the changes of rice quality induced by them. HRT markedly reduced rice quality including high chalkiness, high pasting temperature, high protein content, low total starch content, the increased setback and the elevated consistence. Hence, these results suggested that more attention needs to be focused on the effect of high temperature during reproductive stage on rice quality in further breeding and practice. Moreover, HRT significantly reduced LAI, decreased SLW, declined source-sink ratio (leaf/sink ratio and stem-sheath/sink ratio), hindered the movement of assimilates from stem-sheath to the panicle and shortened the duration of active grain filling. These results suggested that significant reduction in matter translocation after flowering, resulting in the reduced active grain-filling duration and the decreased rice quality, modulated by source-sink characteristics in response to high natural field temperature during reproductive stage. These findings enriched the study of high temperature-stressed rice and served as an important reference for selecting high-quality, heat-tolerant varieties and protecting rice quality under high-temperature conditions.

## Data Availability Statement

The original contributions presented in this study are included in the article/Supplementary Material, further inquiries can be directed to the corresponding author.

## Author Contributions

CC and DT initiated and designed the research, analyzed the data, and wrote the manuscript. DT, WP, and LZ performed the experiments. YJ, AS, CL, and MC revised and edited the manuscript and also provided advice on the experiments. All authors contributed to the article and approved the submitted version.

## Conflict of Interest

The authors declare that the research was conducted in the absence of any commercial or financial relationships that could be construed as a potential conflict of interest.

## Publisher’s Note

All claims expressed in this article are solely those of the authors and do not necessarily represent those of their affiliated organizations, or those of the publisher, the editors and the reviewers. Any product that may be evaluated in this article, or claim that may be made by its manufacturer, is not guaranteed or endorsed by the publisher.
