# Supplementary material for: Response of Source-Sink Characteristics and Rice Quality to High Natural Field Temperature During Reproductive Stage in Irrigated Rice System
Source: Front Plant Sci. 2022 Jul 5;13:911181. doi: 10.3389/fpls.2022.911181 (PMC9294507; doi:10.3389/fpls.2022.911181)
Supplement: Supplementary file 1 [file Data_Sheet_1.docx]

**FIGURE S1 |**  Temperature conditions in two scenarios. A is for scenario A; B is for scenario B. HRT and LRT indicate hot (high temperature) and normal (low temperature) growth season during reproductive stage, respectively. HGT and LGT present hot (high temperature) and normal (low temperature) growth season during grain filling stage, respectively. J565, NJ9108 and J4466 present Jing 565, Nanjing 9108, and Jing liangyou 4466, respectively. The red boxes indicated the significant difference of temperature between two contrasting natural field conditions.

**FIGURE S1**


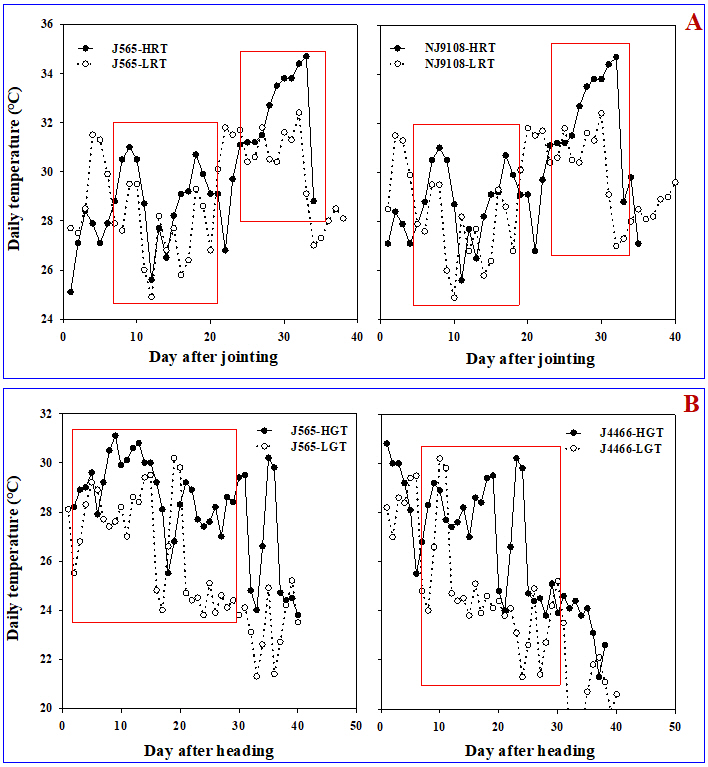


| **TABLE S1 \|** Growth duration and mean temperature during different phenological stages in different situations. HRT and LRT indicate hot (high temperature) and normal (low temperature) growth season during reproductive stage, respectively. HGT and LGT mean hot (high temperature) and normal (low temperature) growth season during grain filling stage, respectively. | | | | | | | | | | |
| --- | --- | --- | --- | --- | --- | --- | --- | --- | --- | --- |
| Scenario | | Treatment | Sowing date (year/m/d) | Jointing date (year/m/d) | Heading date (year/m/d) | Maturity (year/m/d) | VT (℃) | RT (℃) | GT (℃) | GT20 (℃) |
| A | J565-HRT | | 2017/4/15 | 2017/6/26 | 2017/7/28 | 2017/9/28 | 23.8 | 29.7 | 25.9 | 29.0 |
|  | J565-LRT | | 2018/4/19 | 2018/6/23 | 2018/7/31 | 2018/10/4 | 23.8 | 29.1 | 25.8 | 29.1 |
|  | NJ9108-HRT | | 2017/4/15 | 2017/6/27 | 2017/7/30 | 2017/9/29 | 23.8 | 29.9 | 25.7 | 29.0 |
|  | NJ9108-LRT | | 2018/4/19 | 2018/6/25 | 2018/8/4 | 2018/10/6 | 23.9 | 29.2 | 25.5 | 29.0 |
| B | J565-HGT | | 2018/4/19 | 2018/6/23 | 2018/7/31 | 2018/10/4 | 23.8 | 29.1 | 25.8 | 29.1 |
|  | J565-LGT | | 2018/5/25 | 2018/7/15 | 2018/8/16 | 2018/10/11 | 27.0 | 30.0 | 24.2 | 27.8 |
|  | J4466 -HGT | | 2018/4/22 | 2018/6/18 | 2018/8/12 | 2018/9/18 | 23.6 | 28.9 | 26.6 | 28.3 |
|  | J4466-LGT | | 2018/5/1 | 2018/7/16 | 2018/8/25 | 2018/10/11 | 26.5 | 29.4 | 23.5 | 26.3 |
| VT, RT, GT and GT20 represent average daily temperature during vegetative stage, reproductive stage, grain filling stage and the first 20 days of grain filling stage, respectively. J565, NJ9108 and J4466 mean Jing 565, Nanjing 9108, and Jing liangyou 4466. | | | | | | | | | | |

| **Table S2** Mean solar radiation (MJ m^−2^d^−1^) during different phenological stages in different situations. HRT and LRT indicate hot (high temperature) and normal (low temperature) growth season during reproductive stage, respectively. HGT and LGT mean hot (high temperature) and normal (low temperature) growth season during grain filling stage, respectively. | | | | |
| --- | --- | --- | --- | --- |
| Scenario | Treatme | VR | RR | GR |
| A | J565-HRT | 15.7 | 18.1 | 14.9 |
|  | J565-LRT | 15.7 | 17.9 | 14.9 |
|  | NJ9108-HRT | 15.6 | 17.7 | 14.9 |
|  | NJ9108-LRT | 15.7 | 17.6 | 15.0 |
| B | J565-HGT | 15.7 | 17.9 | 14.9 |
|  | J565-LGT | 16.0 | 20.3 | 13.6 |
|  | J4466 -HGT | 15.9 | 17.6 | 14.0 |
|  | J4466-LGT | 15.6 | 19.6 | 13.1 |
| VR, RR and GR represent mean solar radiation during vegetative stage, reproductive stage and grain filling stage, respectively. J565, NJ9108 and J4466 mean Jing 565, Nanjing 9108, and Jing liangyou 4466. | | | | |
